# Editorial: Novel mechanisms for the treatment of AML before and after transplant

**DOI:** 10.3389/fonc.2022.1063307

**Published:** 2022-10-31

**Authors:** Margaret T. Kasner

**Affiliations:** Department of Medical Oncology, Sidney Kimmel Cancer Center, Thomas Jefferson University, Philadelphia, PA, United States

**Keywords:** AML - acute myeloid leukemia, allogeneic stem cell transplantation, novel therapeutic agents, Maintenance therapy, targeted agents

Acute myeloid leukemia (AML) is a molecularly and cytogenetically complex disease that has proven difficult to treat with a plateau in long-term disease-free survival over the last several decades of less than 30% at 5 years. In the past five years, a number of novel agents and combinations have been FDA approved for the treatment of AML. The understanding of novel mechanisms which can be therapeutically targeted for the treatment of AML continues to expand rapidly. In addition attempts to exploit the immune system as a mechanism treat leukemia is expanding beyond allogeneic hematopoietic stem cell transplantation (HSCT) to check point inhibition and T-cell therapies such as BiTE and CAR-T technology. This has led to an increasing number of clinical trials in the treatment of AML as well as in the area of maintenance therapy after intensive induction or consolidative transplantation.

The goal of this Research Topic was to explore novel pathways in AML which could be exploited for therapy of either upfront or relapse/refractory AML as well as in the maintenance setting including maintenance therapy post allogeneic transplantation.

Seven manuscripts in this topic explore a variety of aspects AML therapy, five of which focus on allogeneic transplantation including maintenance. In the first of the papers that focus on non-transplant therapy, Alotaibi et al. describe a series of cases of AML treated with FLT3 inhibitors which developed BCR-ABL1 mutations. This mutation provides an alternative therapeutic target and identifying this mutation at relapse after FLT3 inhibitor failure should be a priority. In the second paper focusing on relapsed AML therapy, Watts et al. introduce a CD3xCD123 bispecific antibody (APVO436). This paper explored the therapeutic and pharmacodynamic effects of APVO436 in 14 relapsed/refractory AML/MDS patients who failed treatement with hypomehylating agents +/- venetoclax. With single agent use at a range of dose levels, *in vivo* immunomodulatory and anti-leukemic activity was demonstrated *via* T-cell activation as well as a median overall survival of 282 days. This paper supports further investigation of the potential benefits of APVO436.

Five manuscripts focus on allogeneic stem cell transplantation ranging from conditioning regimens through DLI to reviews of maintenance options post-transplant. Wang et al. presented a case study of stem cell transplantation using a decitabine-containing preconditioning regimen in TP53 mutated myelodysplastic syndrome. Nine TP53 mutated patients received decitabine preconditioning followed by myeloablative allogeneic HSCT. 89% of patients were alive and progression free at a median follow up of 42 months. Given the extremely poor prognosis of TP53 mutated myeloid malignancy patients, this preliminary evidence of efficacy by decitabine containing conditioning regimens should be explored in larger populations.

Salhotra and Stein. presented a review article that discussed the role of newer radiation based conditioning regimens such as total marrow irradiation (TMI) in patient with high-risk AML undergoing allogenic HSCT in remission or with active disease. They also discuss mechanisms associated with leukemia relapse and treatment options available after relapse following allo-HSCT using intensified conditioning regimens. This comprehensive review provides background on currently used radiation and chemotherapy preparative regiments as well as describing new techniques to deliver radiation using total marrow and lymphoid irradiation which allow for better disease control and improved clinical outcomes. However, relapse remains the primary cause of transplant failure so they further discuss adoptive immunotherapy and maintenance therapies to improve relapse-free outcomes.

In a brief research report by Stadler et al., the use of preemptive and therapeutic donor lymphocyte infusion (preDLI and tDLI) in relapsed myeloid malignancies is explored when using a novel, quantitative PCR-based high-sensitivity chimerism (hs-chimerism). This technique allows for redicition of relapse more than a month before clinical diagnosis. They compared a historic cohort of patients who received preDLI or tDLI prior to hs-chimerism to 32 patients who received these therapies after. At 2 years from first DLI the hs-chimerism group had a very impressive overall and disease-free survival, early 3 times that of controls. This was a result of both reduced relapse incidence with a larger proportion of patients receiving preemptive DLI as well as decreased non-relapsed mortality. This paper supports the value of hs-chimerism for directing DLI after alloSCT and warrants exploration in larger populations across multiple centers.

The final two manuscripts in the Research topic provide comprehensive reviews of post-transplant maintenance therapy for AML after allogeneic HSCT. Manobianco et al. discuss the mechanisms of treatment being targeted for post-transplant maintenance therapy for AML. This includes a discussion of FLT3 inhibitors, histone deacetylase inhibitors, IDH1 & 2 inhibitors, hypomethylating agents, bcl2 inhibitors, and checkpoint inhibitors. The review includes data for their use as well as a discussion of recruiting clinical trials. Nayak and Chen review these agents as well as hedgehog inhibitors and other immunomodulatory agents such as lenalidomide.

The manuscripts presented in this Research Topic provide hope for novel treatments for acute myeloid leukemia before and after transplant, but also highlight the desperate need for expanding our repertoire to treat this complicated disease with a mortality rate of nearly 70% which has not improved over the last decades despite an array of novel therapies.

## Author contributions

The author confirms being the sole contributor of this work and has approved it for publication.

## Conflict of interest

The author declares that the research was conducted in the absence of any commercial or financial relationships that could be construed as a potential conflict of interest.

## Publisher’s note

All claims expressed in this article are solely those of the authors and do not necessarily represent those of their affiliated organizations, or those of the publisher, the editors and the reviewers. Any product that may be evaluated in this article, or claim that may be made by its manufacturer, is not guaranteed or endorsed by the publisher.

